# Metabolomic fingerprinting based on network analysis of volatile aroma compounds during the forced aging of Huangjiu: Effects of dissolved oxygen and temperature

**DOI:** 10.3389/fnut.2023.1114880

**Published:** 2023-01-16

**Authors:** Na Wang, Lili Zhang, Xuejiao Ren, Shuang Chen, Zhen Zhang

**Affiliations:** ^1^School of Food and Health, Jinzhou Medical University, Jinzhou, Liaoning, China; ^2^State Key Laboratory of Food Science and Technology, Key Laboratory of Industrial Biotechnology of Ministry of Education and School of Biotechnology, Jiangnan University, Wuxi, Jiangsu, China

**Keywords:** Huangjiu (Chinese rice wine), aroma, forced aging, temperature, dissolved oxygen, metabolomics, network analysis

## Abstract

**Introduction:**

Huangjiu is an important Chinese alcoholic beverage, usually prepared from rice. Although its unique flavor improves with prolonged storage in traditional pottery jars, knowledge of the aging mechanism, necessary for commercialization of an optimum product, remains unclear.

**Methods:**

Here, volatile aroma compounds from forced aged samples exposed to different temperatures and oxygen treatments were measured by GC/MS. After retention time alignment and normalization, the peak vectors were compared over storage time using Pearson's correlation, and a correlation network was established. Marker compounds, representative of traditionally aged Huangjiu, were then monitored and compared to similar compounds in the forced aged product.

**Results and discussion:**

Correlation network analysis revealed the following: Temperature had little effect on most aroma compounds; alcohols, acids, and esters all increased with increasing dissolved oxygen, while polyphenols, lactones, and ketones were readily oxidized; aldehydes (e.g., furfural and benzaldehyde) were highly dependent on both temperature and dissolved oxygen. Dynamic changes in the targeted aging-markers showed that a higher initial oxygen concentration intensified the “aging-aroma” of Huangjiu in the early and middle stages of storage. Consequently, careful control of oxygen supplementation and storage temperature could be beneficial in controlling the desirable flavor of Huangjiu in the artificially aged product.

## 1. Introduction

Huangjiu, or Chinese rice wine, as a typical fermented rice wine, is known as Chinese national wine, and “Pottery storage, and more aging, more flavors” is one of its most typical characteristics. Although this well-known phrase “more aging, more flavors” is not completely true for all Huangjius (1, 2), the practice proved that there are significant differences between storage in pottery and stainless-steel tank, and this typical “more aging, more flavors” for Huangjiu can be generally characterized only by stored in pottery, not in stainless-steel tank (1, 3, 4). However, it is why?

The unique flavor of traditional Huangjiu is believed to develop during storage over several years in pottery jars. However, to meet increased demand for alcoholic beverages (5, 6), a relatively short-term industrial storage in stainless-steel tank must be instead of the long-term storage in pottery jars for Huangjiu. Therefore, industrial processes are required for the accelerated flavor development of Huangjiu during storage in stainless-steel tanks.

Temperature and dissolved oxygen are recognized as important storage parameters for Huangjiu (2, 4, 7, 8). It is reported that compared with storage in stainless steel, pottery jars have a better oxygen permeability, and their higher thermal mass is less susceptible to changes in ambient temperature so as to keep a stable and suitable temperature of 5–20°C (1, 2), but the model system studies, including oxygen permeability, are still lacking. Moreover, although changes in volatile aroma compounds, amino acids, sensory characteristics, and developments in analytical methods associated with the aging of Huangjiu have been reported (1–4, 7–17), the overall mechanism has not been studied in detail.

Correspondingly, the effects of temperature and dissolved oxygen, as two key factors of Maillard and oxidation flavor reactions in alcoholic beverages (18), have been studied in e.g., red wine, sake, and beer. Among these studies, forced aging simulations combined with kinetics, chemiomics, flavoromics, sensomics, and metabolomics etc. (18–28), have helped to clarify the aging mechanism and regulation of key aroma compounds (e.g., sotolon, vanillin, benzaldehyde, anthocyanins, ferulic acid, and sulfides) (22, 25, 29–34) in different alcoholic beverages. As the demand for high quality products increases, artificial aging is an attractive technology (35–39). However, to realize its potential, a clear understanding of aging mechanisms and product stability during artificial aging is a prerequisite for the commercialization of the process.

While metabolomics is concerned with characterizing the metabolome (22), metabolomics can be defined as the measurement of time-related changes (usually in a smaller set of metabolites) due to an intervention. To date, metabolomics has been successfully applied to the age-dependent characterization of Huangjiu (14) and other wines (9, 30, 40–43). Additionally, network analysis has proved to be a convenient method for visualizing and identifying key relationships between aroma compounds during the aging of some alcoholic beverages (23, 44, 45).

Here, a system was developed to investigate the effects of temperature and dissolved oxygen on the volatile aroma compounds of Huangjiu during forced aging. The aim was to use network analysis and volatile compound data obtained from the analysis of processed samples using headspace-solid phase microextraction (HS-SPME)-GC/MS to obtain information about the mechanisms of aroma formation during the forced aging of Huangjiu. Aging markers identified in previous studies of Huangjiu aged in pottery jars (14–16) provided a useful reference for metabonomic fingerprinting. The results from this small-scale simulation provide a theoretical reference for the development of the commercial aging of Huangjiu in large-scale stainless-steel tanks.

## 2. Materials and methods

### 2.1. Chemicals and materials

All chemicals were of analytical reagent grade: Anhydrous sodium sulfate, sodium chloride, lactic acid, and sodium hydroxide were purchased from China National Pharmaceutical Group Corp. (Shanghai, China). 2-Octanol [internal standard (IS)], methanol, ethanol, dichloromethane, and all other reagents were from Sigma-Aldrich (Shanghai) Trading Co., Ltd., (Shanghai, China). Pure water (>18.18 MΩ cm, 25°C) was obtained from a Milli-Q purification system (Millipore, Bedford, MA, USA).

Young samples of Huangjiu from the same production year were obtained from Zhejiang Guyuelongshan Huangjiu Co., Ltd. (Shaoxing, China). The characteristics of each sample were as follows: Total sugars (glucose), 3.92 g/L; alcohol, 17.2 % (v/v); total acids (lactic acid), 5.86 g/L; sugar solids, 20.90 g/L; dissolved oxygen [O_2_] before the oxygen treatment, 1.8–2.5 mg/L (OX-Y dissolved oxygen sensor, Shanghai Chunye Instrument Technology Co., Ltd., China); and pH 3.5–4.0.

### 2.2. Forced aging

**Figure 1:**
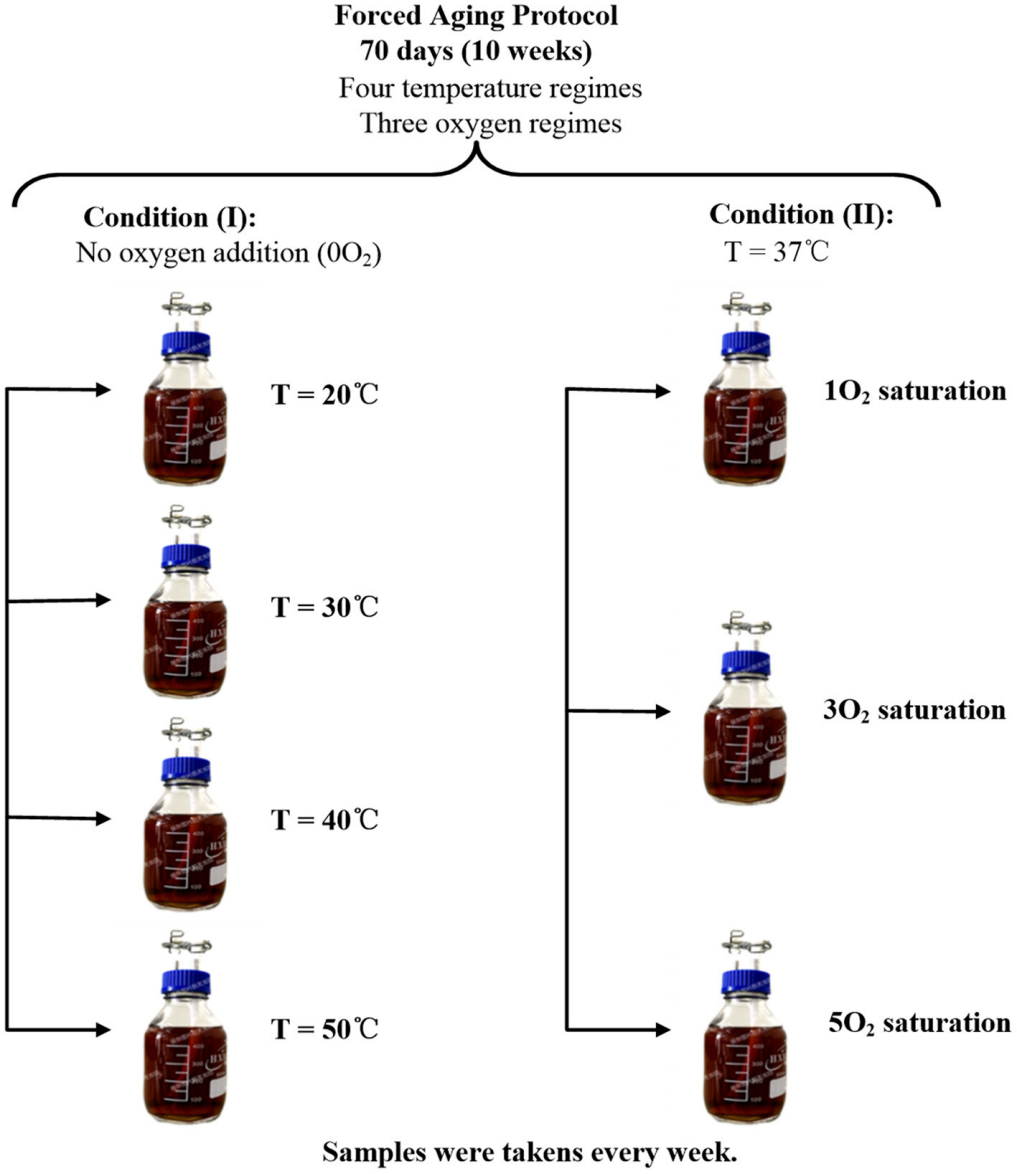
Summary of the forced aging procedure for Huangjiu: All samples were prepared in duplicate; the volume of each sample was 500 mL.

A summary of the experimental procedure used for the forced aging of Huangjiu was given in Figure 1. Two regimes of seven groups were kept for 70 days, and all samples were prepared in duplicate, where the setting of temperature and dissolved oxygen referred to and was modified from the existed researches on beer and other wines (19–21, 25).

Based on this forced-aging protocol, all samples in condition (I) were placed in a magnetic stirring water-bath with four different temperatures of 20, 30, 40, and 50°C, and no oxygen was added (0O_2_) during this forced-aging 70 days (19–21). Correspondingly, all samples in condition (II) kept a constant 37°C temperature (25). Meanwhile, samples for condition (II) were labeled 1, 3, and 5O_2_, where 1–5 denotes the number of oxygen treatments. Oxygen saturation was achieved by stirring the sample for ~1 h until the oxygen concentration reached 8–9 mg/L (OX-Y oxygen sensor) (19–21): 1O_2_ was saturated on day 35; 3O_2_ was saturated on days zero, 28, and 56; 5O_2_ was saturated on days zero, 14, 28, 42, and 56 (Figure 1). All apparatus was cleaned and sterilized prior to use. Pyrex GL 45 media bottles were each filled with Huangjiu (each sample 500 mL), GL45 screw cap twin hose connectors, stainless steel vent pipes, hoses, and clamps were fitted, and the samples were placed in a constant temperature water bath at 90°C for 30 min to sterilize the media. When cool, samples 3 and 5O_2_ were subjected to oxygen saturation, all vent pipe hoses were clamped, and the samples were treated according to the conditions given in Figure 1.

Samples taken at predetermined times were stored at −20°C until required for analysis. To reduce the GC/MS analytical burden, samples from the duplicate batch were selected at random and used as a crosscheck validation. Prior to analysis, all samples were subjected to a simple odor test to verify that the Huangjiu was viable. Additionally, the initial volume of 500 mL would be less and less with sampling, so we took a cross-sample in duplicate samples every other week to reduce the influence of sampling on the sample volume reduction.

### 2.3. HS-SPME-GC/MS analysis of volatile compounds

The forced-aging experimental protocol was performed in duplicate for practical reason and some samples were analyzed by GC/MS on the replicate trial. Referring to our existed studies (14, 46), two milliliters of Huangjiu samples, 8 mL of pure water with 3 g of sodium chloride and 10 μl of IS (2-O, 68.344 ppm) were mixed into 20 mL screw-capped vial for GC/MS analysis. An automatic headspace (HS) sampling system with an Agilent GC68905975MSD was used for extraction of volatile compounds in samples, and DBFFAP column was used for the separations. The oven temperature was initially held at 50°C for 2 min, then raised at 5°C/min to 230°C for 15 min. Data acquisition was in the selective ion monitoring (SIM) mode (ionization energy = 70 eV).

### 2.4. Data analysis

#### 2.4.1. Pre-processing

Feature detection, retention time (RT) correction, and preliminary statistical analyses were carried out using XCMS online (XCMSOnline version 2.2.5; XCMS version 1.47.3; CAMERA version 1.26.0, the Scripps Center for Metabolomics, La Jolla, CA; https://xcmsonline.scripps.edu). Raw data conversion, GC/MS spectra processing, set parameters, etc., were described in Yu et al. (9). All chromatograms were simultaneously analyzed under the same conditions. From seven different conditions of the forced aging procedure (Figure 1), the multi-group job was selected, and the seven groups were classified.

#### 2.4.2. Optimization of candidate features

Using the extracted features from XCMS-online software as the Y-Variable, a bivariate Pearson's correlation was performed. The marked significant Y-variables, which were the optimized candidate features based on a false discovery rate≤ 0.05, were selected as candidate features for network analysis and qualitative and quantitative analyses. To further simplify these analyses and delete the corresponding features of the IS and ethanol, the m/z and RT of all extracted features were compared in METLIN (https://metlin.scripps.edu) and the NIST 05 library on the GC/MS workstation (Agilent Technologies Inc., USA).

#### 2.4.3. Identification and quantification of candidate features in network analysis

The identification of candidate features in network analysis directly referred to the existing our study (14): The aligned matrix (/.xlsx) from XCMS-Oline software (https://xcmsonline.scripps.edu) consists of all the exported metabolite features-putative identifications through METLIN standard database matching, which are defined as ions with unique m/z and RT values (Supplementary material). Finally, MS features were validated by (i) crosschecking their presence in the raw data, (ii) comparing their features with those present in the NIST 05 (matching degree %) and NIST 98 MS library (http://webbook.nist.gov), and (iii) comparing with the existing identifications (14–17, 46).

Finally, all identified volatile compounds, including unknowns, were directly quantified using HS-SPME-GC/MS.

#### 2.5. Statistical methods

Bivariate Pearson's correlation was carried out using IBM SPSS Statistics for Windows, version 19.0 (IBM Corp., Armonk, NY USA). Network analysis of the optimized candidate features was performed with Gephi version 0.9.2 (https://gephi.org/) using the Fruchterman–Reingold algorithm. All box plots and PCA sores plots were exported directly from XCMS-online software. Data trends were graphed with OriginPro 8.5.0 SR1 (The OriginLab Corporation, USA).

## 3. Results and discussion

### 3.1. Preliminary analysis of untargeted SPME-GC/MS-based metabolomics

**Figure 2:**
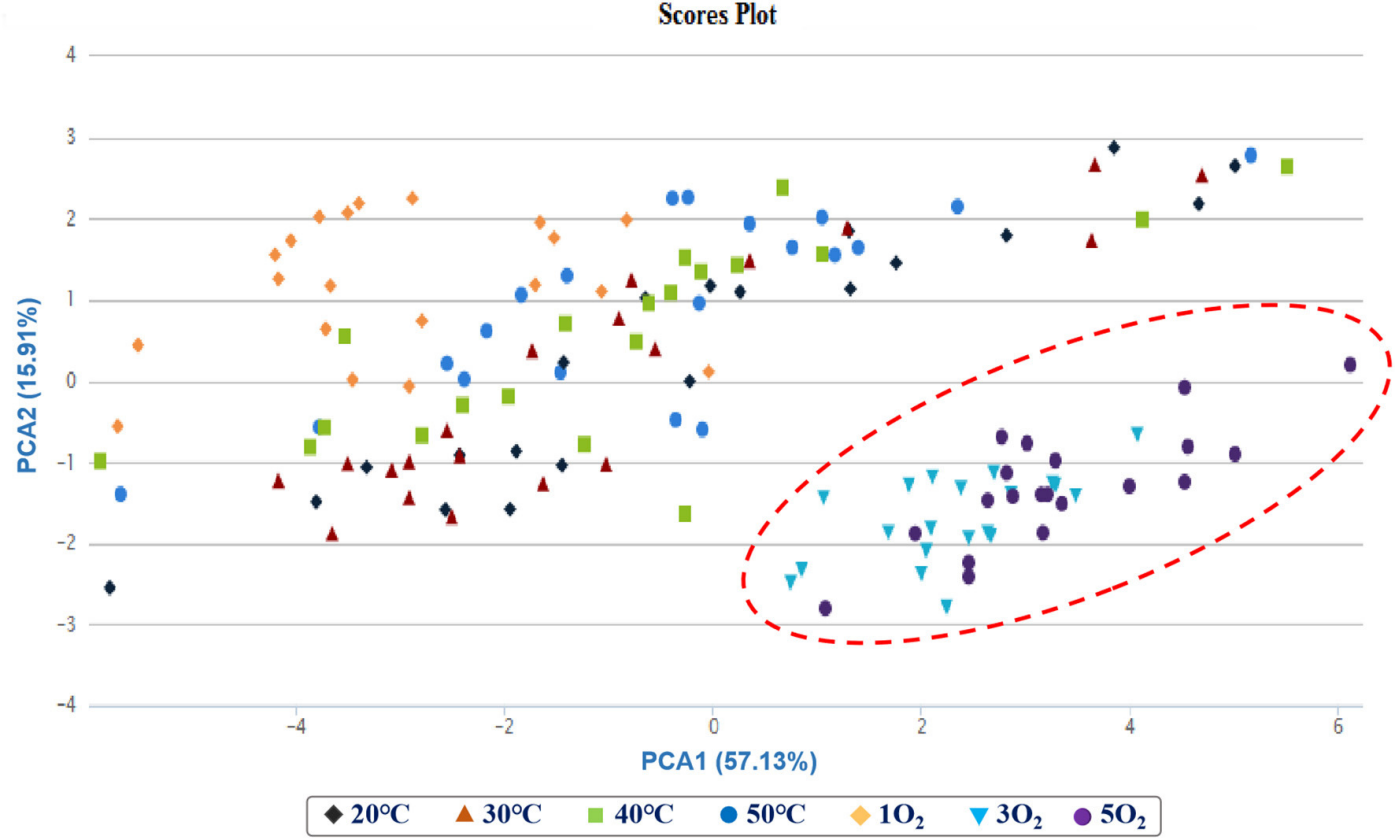
PCA scores plot obtained from the untargeted SPME-GC/MS data for the forced aging samples.

A total of 149 candidate features were extracted from the raw HS-SPME-GC/MS data (Supplementary material) of all 140 Huangjiu samples after pre-processing. Meanwhile, a preliminary analysis of PCA was directly exported from XCMS-online software based on 149 candidate features and 140 Huangjiu samples, and the PCA result (Figure 2) showed that samples 3 and 5O_2_ from condition (II) could be distinguished from all other forced aging treatments based on the first two principal components, PC1 and PC2, representing 57.53 and 15.91% of the total variation, respectively. Compared with all samples from condition (II), 10 samples from condition (I) also showed a wider distribution, and these preliminary results implied that the different temperatures in condition (I) had a significant effect on the aging time (increase or decrease) of Huangjiu, but frequent oxygenation [e.g., 3 and 5O_2_ in condition (II)] promoted changes in the volatile aging compounds. However, this PCA result was to be verified and improved.

### 3.2. Network analysis fingerprinting

Based on the PCA result above, in order to verify the influence of temperature and dissolved oxygen in our experiment further, we decided to further use network analysis method to quickly extract and identify compounds with similar trends under different conditions.

Following repeated comparison and screening (*m/z* and RT) of the 149 candidate features using the METLIN and NIST 05 databases, peaks due to the IS (2-octanol) and ethanol were deleted. A bivariate Pearson's correlation and network analysis of the remaining 112 characteristic peaks were then used to determine the characteristic peaks and markers for the effects of oxygen and temperature on volatile compounds during the forced aging.

**Figure 3:**
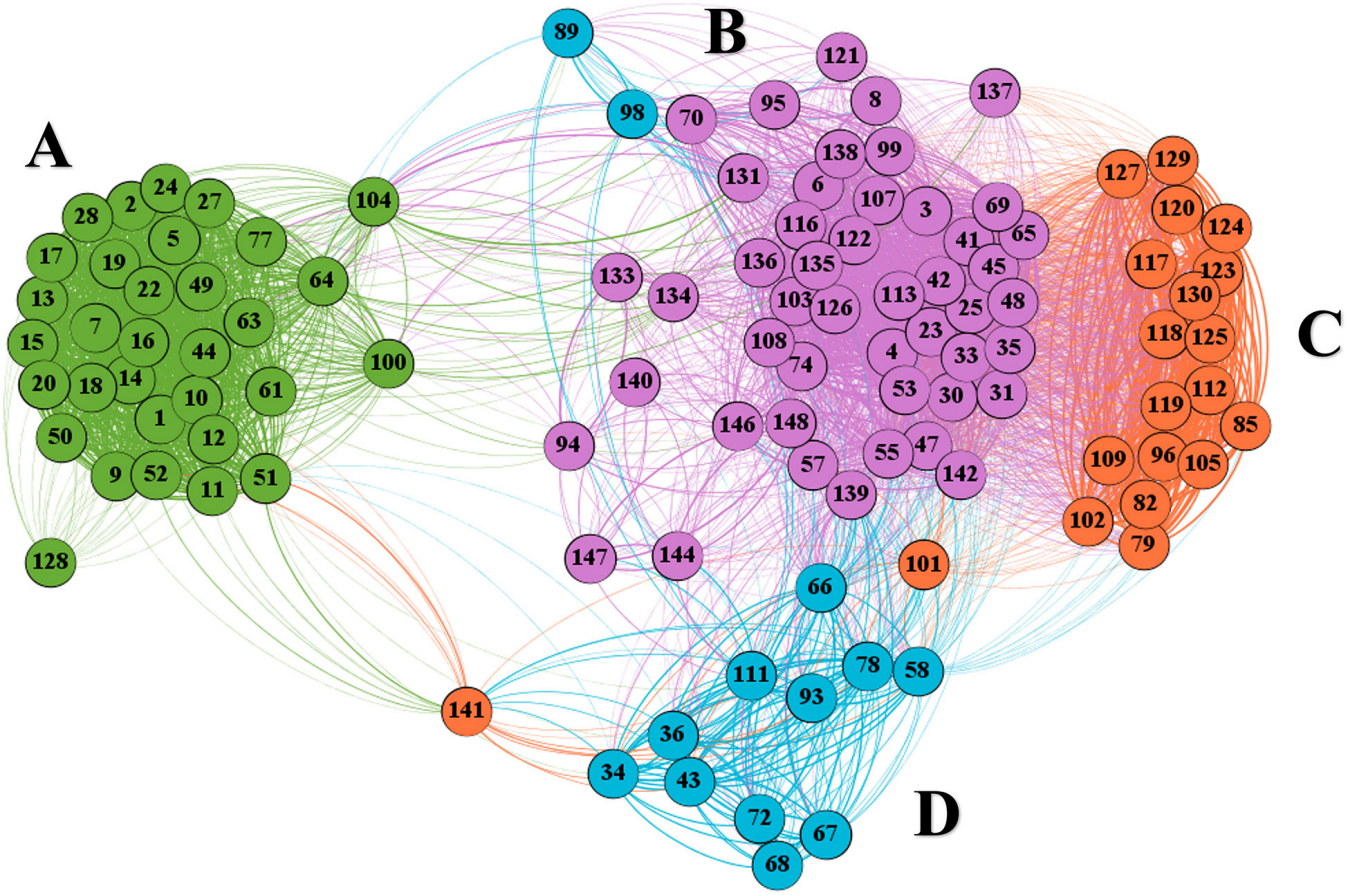
**(A–D)** Network analysis shows the correlated characteristic peaks obtained from the untargeted GC/MS data: The nodes represent the characteristic peaks, while the different densities of the connecting edges indicate the relative strength of the associations. The number is the code of characteristic peaks, and the different color regions represent different conditions (temperature and oxygenation) with time.

Figure 3 showed the network analysis map obtained from untargeted GC/MS data: The 112 characteristic peaks were represented as nodes, while the magnitude of the associations could be visualized from the densities of the interconnecting edges; the nodes were divided into four regions (A–D), each representing those characteristic peaks with significant positive correlations and similar changes during this forced aging. Although these characteristic peaks in the same color region had the significant positive correlations and similar changes during this forced aging, they could come from different compounds.

**Figure 4:**
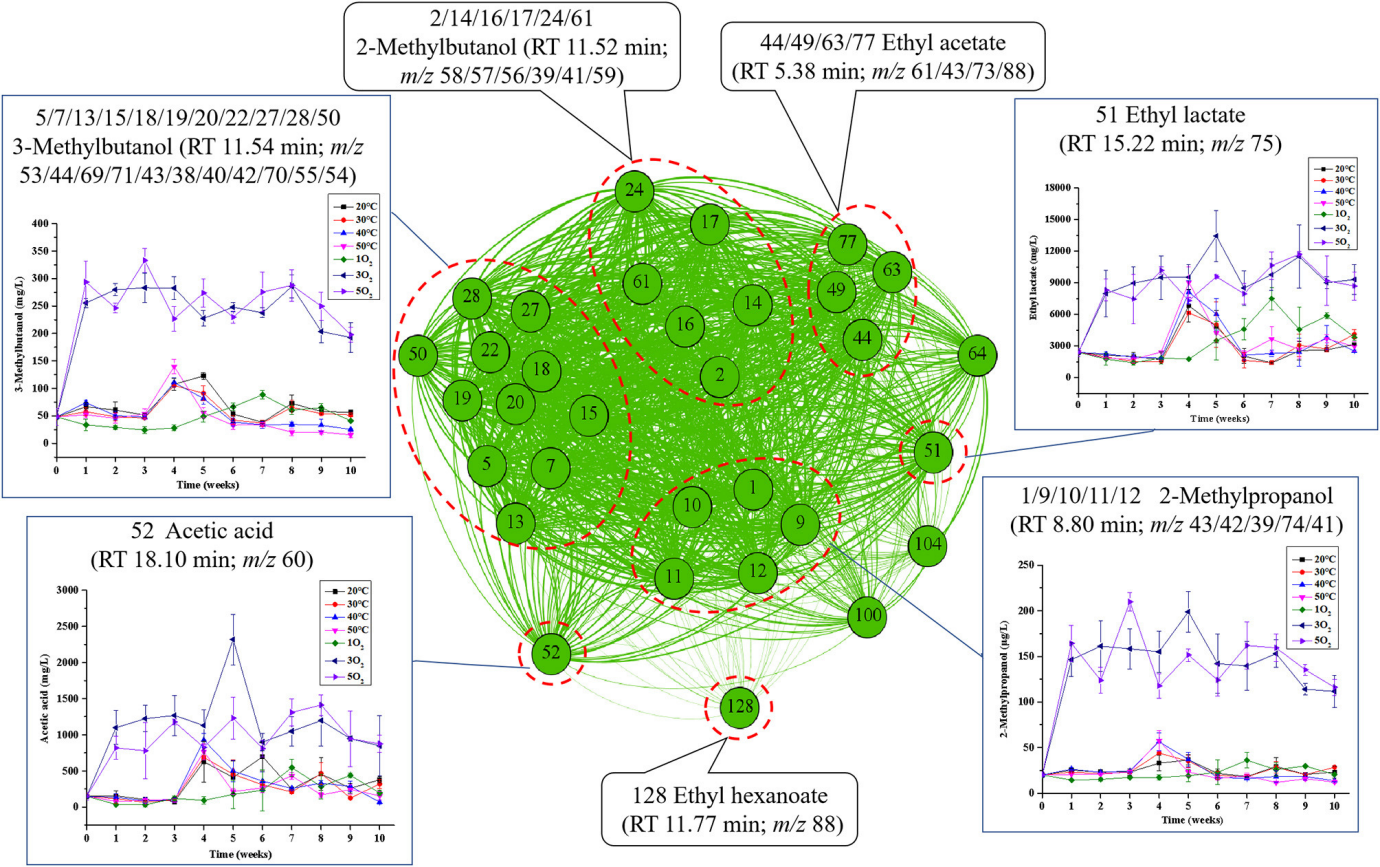
Network analysis map showing the associations between the characteristic peaks in region A: Inset charts show changes in the concentrations of marker compounds for each treatment condition (temperature and oxygenation) with time. The nodes represent the characteristic peaks, while the different densities of the connecting edges indicate the relative strength of the associations in this region. The number in front is the code of characteristic peaks, corresponding with those in Figure 3, and the number in the back is the observed features (m/z).

**Figure 5:**
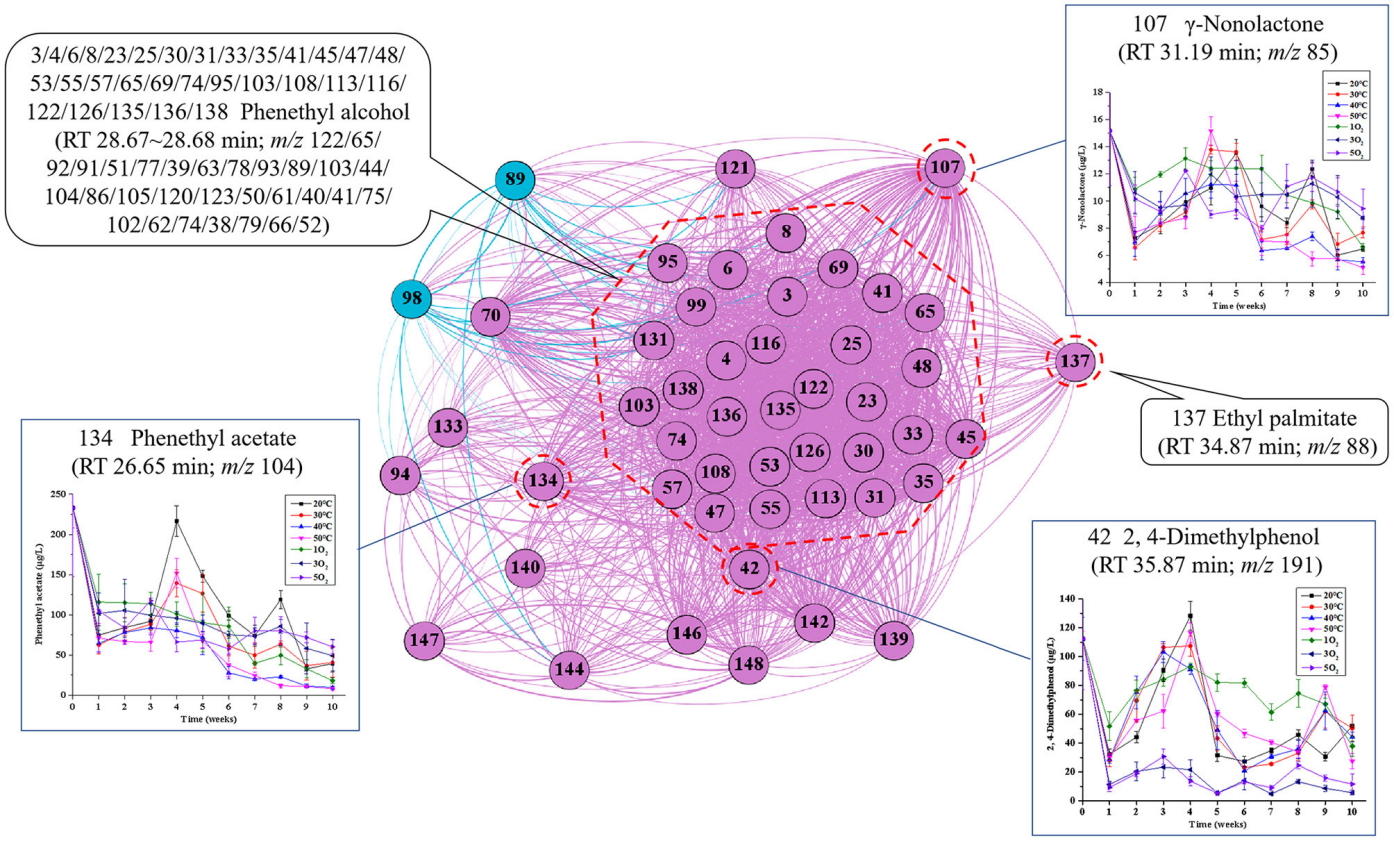
Network analysis map showing the associations between the characteristic peaks in region B: Inset charts show changes in the concentrations of marker compounds for each treatment condition (temperature and oxygenation) with time. The nodes represent the characteristic peaks, while the different densities of the connecting edges indicate the relative strength of the associations in this region. The number in front is the code of characteristic peaks, corresponding with those in Figure 3, and the number in the back is the observed features (m/z).

**Figure 6:**
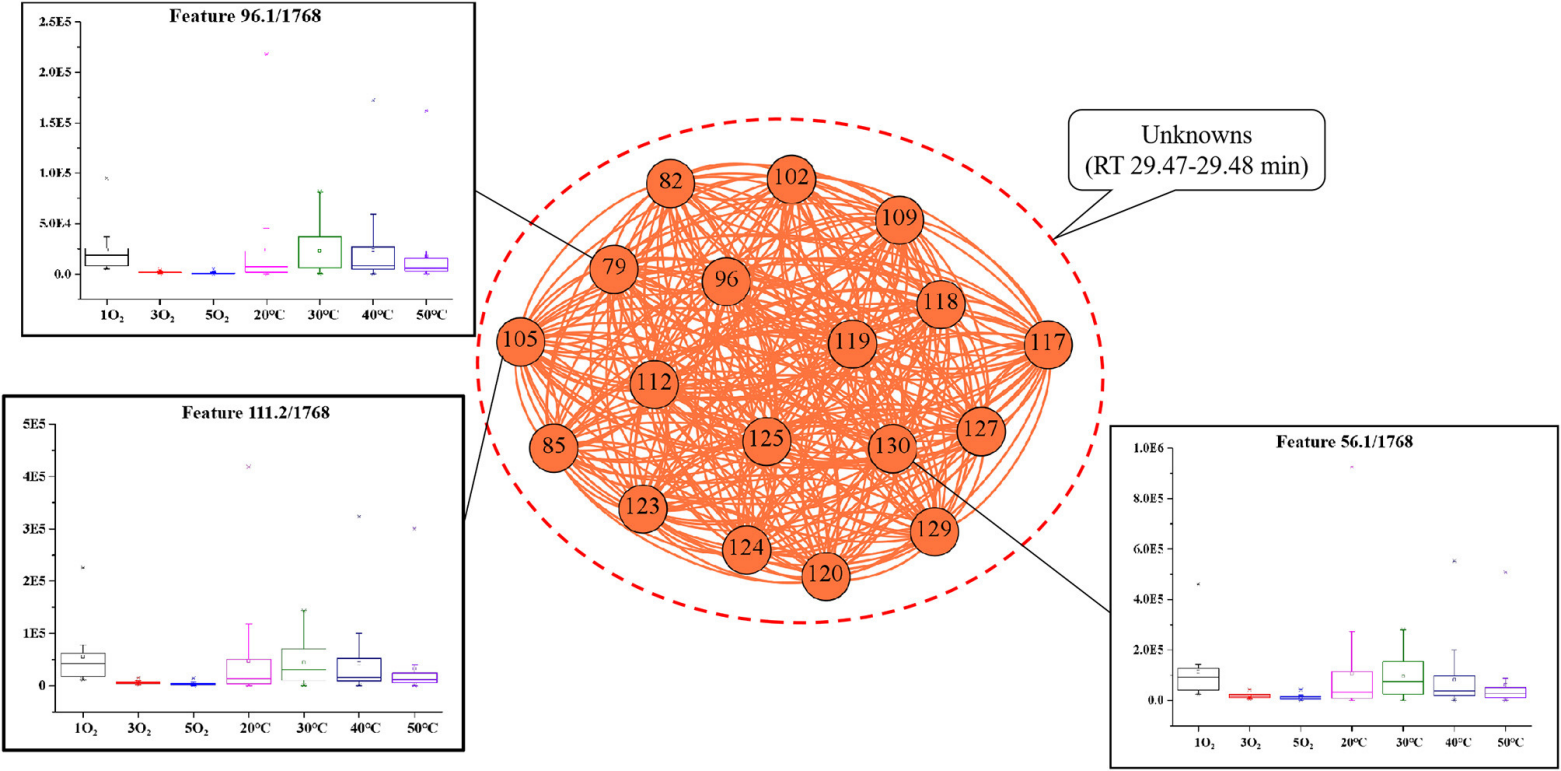
Network analysis map showing the associations between the characteristic peaks in region C: Inset box plots show the abundance for each treatment condition (temperature and oxygenation). The nodes represent the characteristic peaks, while the different densities of the connecting edges indicate the relative strength of the associations in this region. The number in front is the code of characteristic peaks, corresponding with those in Figure 3, and the number in the back is the observed features (m/z).

**Figure 7:**
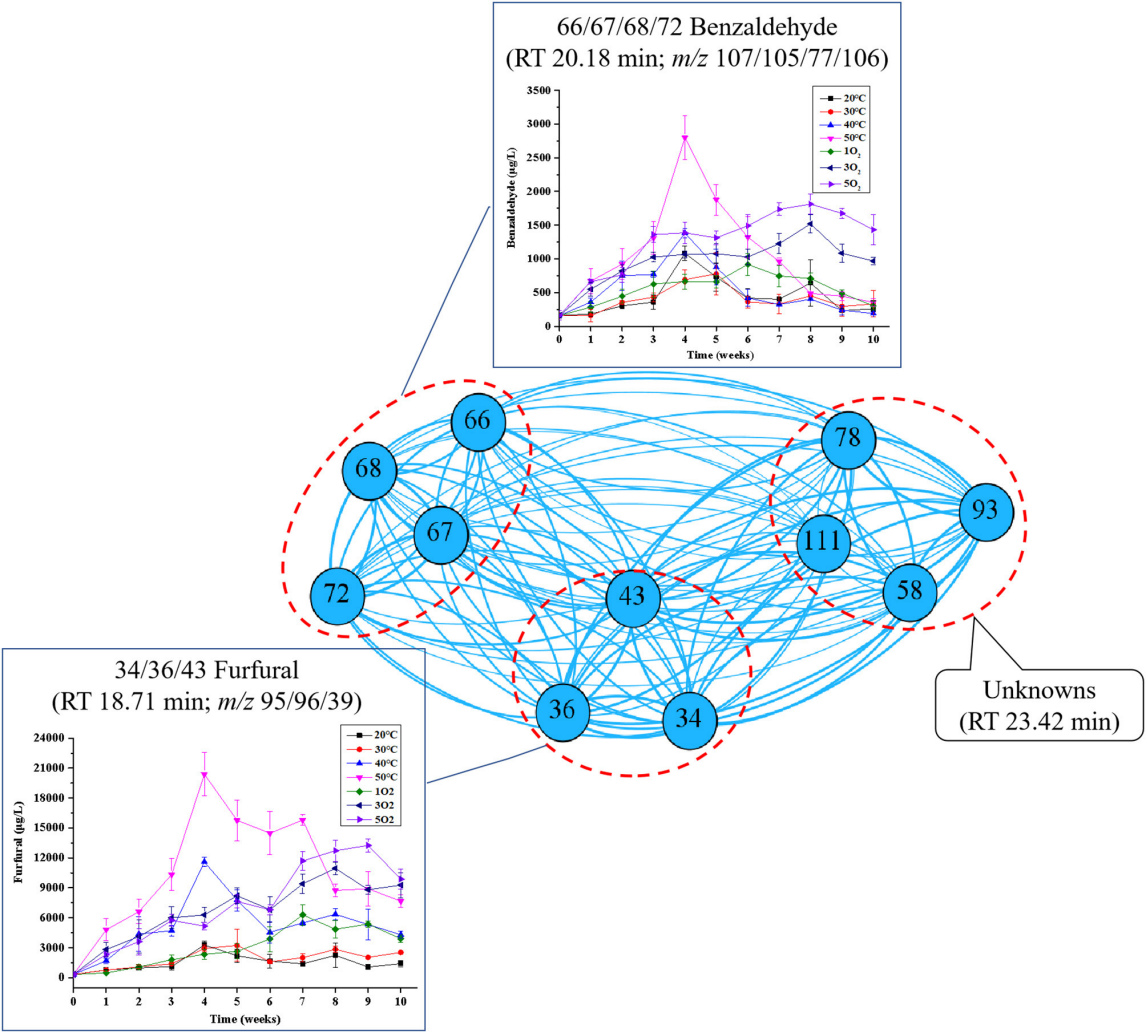
Network analysis map showing the associations between the characteristic peaks in region D: Inset charts show changes in the concentrations of marker compounds for each treatment condition (temperature and oxygenation). The nodes represent the characteristic peaks, while the different densities of the connecting edges indicate the relative strength of the associations in this region. The number in front is the code of characteristic peaks, corresponding with those in Figure 3, and the number in the back is the observed features (m/z).

Based on the preliminary identification and quantification of some nodes, network analysis was used to further visualize the relationships and differences among all volatile aging compounds present in each region (A-D), as well as changes in their concentrations over time (Figures 4–**7**).

#### 3.2.1. Correlation network analysis of region A

As shown in Figure 4, about 69% of all the candidate features in region A were identified as alcohols (e.g., 2- and 3-methylbutanol, 2-methylpropanol), followed by ethyl acetate, ethyl lactate, ethyl hexanoate, and acetic acid. Some common characteristics and trends were observed for samples from conditions (I) and (II). For example, the content of 2-methylpropanol, 3-methylbutanol, ethyl lactate, and acetic acid was significantly higher in sample treatments 3 and 5O_2_ compared with 1O_2_. Oxygen supplementation at week 5 (day 35, 1O_2_) showed an increase in marker compounds, which then decreased in the latter stages of storage. In contrast, the four different temperatures of condition (I) had little effect on the amount of volatile aroma compounds. Hence, the results suggested that amounts of the volatile marker compounds in region A were dependent on dissolved oxygen and not readily oxidized.

Previously we found that amounts of 2- and 3-methylbutanol and 2-methypropanol showed significant decreasing trends during the aging of Huangjiu, and these compounds were representative aging markers, especially for the short-aged wine (14–16). Here, 3-methylbutanol and 2-methylpropanol showed some association with 3 and 5O_2_, but the decreasing trend fluctuated greatly, especially in sample 5O_2_. In a study on the aroma of fortified wine, a high initial oxygen concentration increased the concentration of aroma compounds during aging (21). Based on these findings, we speculated that the interval and frequency of oxygenation during aging could increase overall alcohol production. Since a higher oxygenation frequency also increased the concentration of other aroma compounds, we proposed that its introduction in the early stage of aging would be beneficial for the flavor of Huangjiu.

The organic acids in wine can be formed from the oxidation of alcohols and aldehydes and the hydrolysis of esters, while amounts of 2- and 3-methylbutanol, 2-methylpropanol, acetic acid, ethyl acetate, ethyl hexanoate, ethyl lactate mainly originated from the raw materials and fermentation process of Huangjiu. Consequently, the concentrations of these flavor compounds during fermentation can determine their individual reactivities (23). Since the hydrolysis of esters largely determined concentrations of the corresponding organic acids and alcohols in the aging process, it was reasonable to assume that the above three alcohols, acetic acid, and three ethyl esters occurred in the same environment of region A.

#### 3.2.2. Correlation network analysis of region B

Most of the candidate features in region B were identified as phenethyl alcohol, with lesser amounts of γ-non-alactone, ethyl palmitate, 2,4-dimethylphenol, phenethyl acetate, and other unknowns (Figure 5). Contrary to region A, the interval and frequency of oxygenation (3 and 5O_2_) decreased the concentration of these volatile aroma compounds.

Many phenolic compounds have antioxidant properties and are readily oxidized during aging. Correspondingly, the content of 2,4-dimethylphenol following 1O_2_ supplementation was significantly higher than that with 3 and 5O_2_, suggesting that a higher initial concentration of dissolved oxygen increased the oxidation of phenolic compounds during aging. The presence of both phenethyl acetate and phenethyl alcohol in the same region also indicated that the ester was mainly derived from the reaction of the parent alcohol with acetic acid.

The fatty acid ester ethyl palmitate is readily hydrolyzed to palmitic acid and ethanol in an acidic environment (47). Previously we reported that the relatively high concentration of ethyl palmitate in young Huangjiu decreased throughout the aging process, while the total acid content showed the opposite trend (14, 16).

Since the volatile compounds (including unknowns) in region B were readily oxidized, dissolved oxygen had a greater influence on the aging aroma of Huangjiu than temperature.

#### 3.2.3. Correlation network analysis of region C

Compared with the box-plots derived from the untargeted GC/MS data (Figure 6), all the candidate features in region C were pre-confirmed as the same component (RT 29.47–29.48 min). The identity of the unknown could not be confirmed because of the limited mass accuracy of the single quadrupole mass spectrometer used. Inspection of the inset box plots of Figure 6 shows that the abundance of some unknown compounds (i.e., nodes 79, 105, and 130) approached zero at 3 and 5O_2_ in condition (II). This implied that the molecular species were easily oxidized, and the aroma compounds in region C had similar characteristics to those of region B.

#### 3.2.4. Correlation network analysis of region D

As shown in Figure 7, benzaldehyde was the most abundant volatile compound, followed by an unknown and furfural. Benzaldehyde can be formed by the oxygenation of its parent alcohol, which could account for the higher concentrations of the aldehyde found in samples 3 and 5O_2_ compared with 1O_2_. The highest concentration of benzaldehyde and the most significant change occurred in samples at 50°C and 5O_2_, respectively, which agreed with the reported oxygen and temperature dependence of benzaldehyde (and sotolon) during Port wine aging (23). The identification of benzaldehyde may also be useful in unraveling the connection between the Maillard mechanism and oxidation during the aging of Huangjiu. Temperature also had a significant effect on the formation of furfural in region D during aging.

Maillard reactions are regarded as the major pathways of furan formation. Furans are responsible for the key “caramel-like” aroma in aged wine (17), and their formations are believed to occur *via* Maillard reactions and oxidation (21, 23). The aldehyde substituted furan, furfural, is a common flavor compound in wine, and its formation was shown to be significantly affected by temperature during the aging of a fortified wine (21). Here the concentration of furfural increased with increasing temperature (40 and 50°C) and dissolved oxygen (3 and 5O_2_). Although the effect of condition (II) was much less than that of condition (I), furfural showed a strong association between time and dissolved oxygen (*r* values: 0.826^**^, 0.802^**^, and 0.819^**^ at 1, 3, and 5O_2_, respectively). Hence, dissolved oxygen may also play an important role in the formation of furfural.

#### 3.2.5. Network analysis fingerprinting: Summary of effects

Temperature had little effect on the aging-aromas in regions A, B, and C, but the alcohols, acids, and esters in region A were highly dependent on oxygen. Although they were not easily oxidized, their concentrations increased with increasing dissolved oxygen. In contrast, the polyphenols, lactones, ketones, and unknowns in regions B and C were easily oxidized, and their concentrations were significantly affected by dissolved oxygen. The effects of both temperature and dissolved oxygen on the formation of the key marker compounds, furfural, and benzaldehyde, in region D, confirmed the importance of these parameters during the aging of Huangjiu (15, 17). The interval and frequency of oxygenation also increased their concentrations.

### 3.3. Effects of dissolved oxygen and temperature on key aging-markers during the forced-aging of Huangjiu

To compare the effects of forced-aging with the natural process, six aging-markers (2-phenyl-2-butenal, ethyl phenylacetate, 3-methylbutyric acid, acetophenone, isoamyl acetate, and γ-butyrolactone) were selected from previous investigations of Huangjiu (14–16). Although these compounds could not be extracted by the untargeted GC/MS-based network analysis method used in this study, they were considered to be important markers for the aging status of Huangjiu (15, 16).

**Figure 8:**
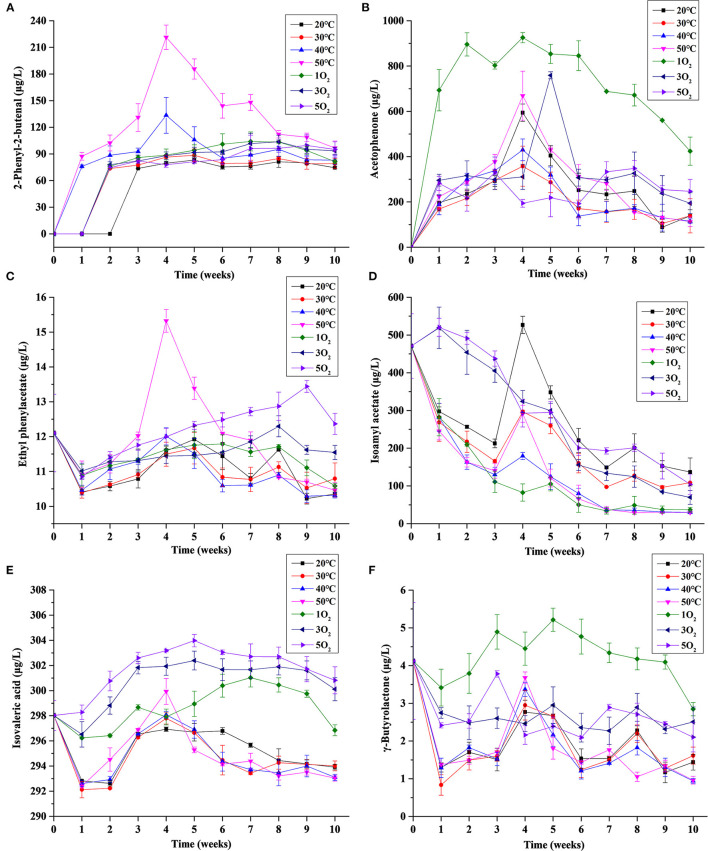
Effects of temperature and dissolved oxygen on the concentrations of key marker compounds during the forced aging of Huangjiu: **(A)** 2-Phenyl-2-butenal; **(B)** acetophenone; **(C)** ethyl phenylacetate; **(D)** isoamyl acetate; **(E)** isovaleric acid (3-methylbutanoic acid); and **(F)** γ-butyrolactone at different conditions.

Figure 8 showed the influence of temperature and dissolved oxygen on the key marker compounds in Huangjiu.

#### 3.3.1. Aromatic marker compounds

The aromatic compounds (2-phenyl-2-butenal, ethyl phenylacetate, acetophenone; Figures 8A–C) showed an initial increasing trend for most conditions during the forced aging process. These compounds, together with benzaldehyde, are characteristic markers of the long-aged product (10–15 years) (14–16) and are, therefore, indicators of high-quality Huangjiu. However, the concentration of these markers all decreased after 4 or 5 weeks for samples under condition (I), and this trend increased with increasing temperature (Figures 8A–C). In the presence of oxygen, the initial increasing trend persisted until weeks 8–9 (especially 3 and 5O_2_). Compared with the traditional aging in pottery jars, the decrease in the three aromatic aging-markers after 4 or 5 weeks under the condition (I) represented the largest difference. This difference could be attributed to insufficient dissolved oxygen and poor oxygen permeability in the forced aging process.

In addition, the trends for each treatment condition showed that the effect of temperature on 2-phenyl-2-butenal and ethyl phenylacetate was greater than that of dissolved oxygen (Figure 8A), and so was ethyl phenylacetate, while dissolved oxygen had a greater effect on acetophenone, phenethyl alcohol, and phenethyl acetate (Figure 5).

#### 3.3.2. Non-aromatic marker compounds

The overall decreasing trends in the concentrations of isoamyl acetate showed a spiked increase for the condition (I) sample at week 4, the magnitude of which decreased with increasing temperatures (Figure 8D), and this agreed with a previous investigation of Huangjiu (15). Higher initial concentrations of isoamyl acetate, and a greater decreasing trend, were observed in samples subject to increased oxygenation (2 and 3O_2_), and this mirrored the behavior of 3-methlybutaol and acetic acid in region A of network analysis (Figure 4).

The highest amounts of isovaleric acid (Figure 8E) were also found in samples with the highest oxygenation treatments. This was similar to the behavior of compounds in region A of network analysis, and its effect was greater than that of temperature. Hence, isoamyl acetate and isovaleric acid, together with the volatile compounds identified in region A of network analysis (Figure 4), constitute potential markers for the forced aging process of Huangjiu.

#### 3.3.3. Lactone marker compounds

Lactones are cyclic organic esters of the corresponding carboxylic acids, and their formations in wine are reported to be sensitive to temperature (23). Here a slightly higher concentration of γ-butyrolactone (Figure 8F) existed at 50°C compared with the other temperature treatments. This followed the behavior of γ-non-olactone (Figure 5), but the trends were not clear. The concentration of γ-butyrolactone was highest at 1O_2_ (Figure 8F), compared with 3 and 5O_2_, indicating that the lactone was readily oxidized during the forced aging process. Hence dissolved oxygen had a greater influence on γ-butyrolactone than temperature. However, the regulation of dissolved oxygen on a commercial scale, and optimization of γ-butyrolactone and γ-non-olactone formation, may require development.

#### 3.3.4. Other marker compounds

Although the key marker compounds ethyl butyrate and ethyl 3-methylbutyrate were expected, they were only detected in some samples at 50°C, and their concentrations were very low. Consequently, their absence/low concentration suggests that the forced aging procedure may require further development/validation.

## 4. Conclusions

Metabonomic fingerprinting based on network analysis of untargeted GC/MS volatile compound data, and quantitative analysis of targeted marker compounds, was successfully applied to determine the effects of dissolved oxygen and temperature on the forced aging aroma of Huangjiu. Alcohols, acids, and esters all increased with increasing dissolved oxygen, while polyphenols, lactones, and ketones were readily oxidized; aldehydes (e.g., furfural and benzaldehyde) were highly dependent on both temperature and dissolved oxygen. Dynamic changes of six marker compounds during the laboratory scale forced aging of Huangjiu identified potential limitations in aroma generation for the product stored in large-scale stainless-steel tanks. Higher initial concentrations of oxygen, together with supplementation in the middle stages, favored the generation of stronger aging aromas. While higher temperatures promoted the concentration of some key marker compounds, this observation required verification and optimization on a commercial scale. Consequently, here the model system was to be further developed, including a further study on the kinetics of the identified aging markers, but this study would provide a theoretical reference for the development of high quality Huangjiu using modern production methods.

## Data availability statement

The original contributions presented in the study are included in the article/Supplementary material, further inquiries can be directed to the corresponding authors.

## Ethics statement

Ethical review and approval were waived for this study, because Huangjiu samples used in the study are consumed in daily life.

## Author contributions

NW: methodology, data curation, and writing—original draft preparation. SC and NW: writing—review and editing. LZ, XR, and ZZ: supervision and resources. NW, LZ, XR, and ZZ: project administration and funding acquisition. All authors have read and agreed to the published version of the manuscript.

## References

[1] Wang YX. Elementary introduction of storematurity of yellow rice wine. Liquor Making Sci Technol. (1999) 3:66–7.

[2] Yang GJ. Aging of Chinese rice wine. Liquor Making Sci Technol. (2006) 6:74–6.

[3] Luo SM Ni B Zhang QW. Effects of different storage methods on the flavor and organic acids of Huangjiu. Liquor Making Sci Technol. (2021) 2021:78–84. 10.13746/j.njkj.2020198

[4] Hu J Chi GH. Investigation on the orignal of aging-aromas in Huangjiu. Liquor Making Sci Technol. (2012) 2:30–2.

[5] Li ZF Kong HC Liu YF Liu YF Chen J. Future foods: Opportunity and challenge. J Chin Inst Food Sci Technol. (2022) 22:1–13. 10.16429/j.1009-7848.2022.04.001

[6] Davis KF Gephart JA Emery KA Leach AM Galloway JN D'Odorico P. Meeting future food demand with current agricultural resources. Global Environ Chang. (2016) 39:125–32. 10.1016/j.gloenvcha.2016.05.004

[7] Han X Mao J Huang GD. Effect of trace ventilation on flavoring substances and free amino acids in Chinese rice wine during storage. Food Sci. (2013) 34:123–7.

[8] Zhou XD Wang RQ Shen QT Zhu YY Wu YH Tian RG. Study on the transfer rate of oxygen in pottery jar of Shaoxing-rice wine. Acta Agri Boreali-occidentalis Sinica. (2016) 25:950–4.

[9] Yu H Zheng D Xie T Xie J Tian H Ai L. Comprehensive two-dimensional gas chromatography mass spectrometry-based untargeted metabolomics to clarify the dynamic variations in the volatile composition of Huangjiu of different ages. J Food Sci. (2022) 87:1563–74. 10.1111/1750-3841.16047 35262917

[10] Yang Y Ai L Mu Z Liu H Yan X Ni L. Flavor compounds with high odor activity values (OAV >1) dominate the aroma of aged Chinese rice wine (Huangjiu) by molecular association. Food Chem. (2022) 383:132370. 10.1016/j.foodchem.2022.132370 35183960

[11] Wei Z Zhang J Shao W Wang J. Fabrication and application of three-dimensional nanocomposites modified electrodes for evaluating the aging process of Huangjiu (Chinese rice wine). Food Chem. (2022) 372:131158. 10.1016/j.foodchem.2021.131158 34601421

[12] Ma Y Guo S Zhang J Xu Y Wang D. Kinetic modeling of ethyl carbamate formation from urea in Huangjiu during storage. Food Control. (2021) 129:108249. 10.1016/j.foodcont.2021.108249

[13] Xu JF Zhang FJ. Changes of aroma substances, taste characteristics and surface tension during the aging process of Chinese rice wine. China Brew. (2018) 1:41–4. 10.11882/j.issn.0254-5071.2018.01.009

[14] Wang N Chen S Zhou ZM. Age-dependent characterization of volatile organic compounds and age discrimination in Chinese rice wine using an untargeted GC/MS-based metabolomic approach. Food Chem. (2020) 325:126900. 10.1016/j.foodchem.2020.126900 32387958

[15] Wang N Zhou ZM Chen S. Aging status characterization of Chinese rice wine based on key aging-marker profiles combined with principal components analysis and partial least-squares regression. Eur Food Res Technol. (2020) 246:1283–96. 10.1007/s00217-020-03488-x

[16] Wang N Chen S Zhou ZM. Characterization of volatile organic compounds as potential aging markers in Chinese rice wine using multivariable statistics. J Sci Food Agric. (2019) 99:6444–54. 10.1002/jsfa.9923 31294830

[17] Chen S Wang CC Qian MC Li Z Xu Y. Characterization of the key aroma compounds in aged Chinese rice wine by comparative aroma extract dilution analysis, quantitative measurements, aroma recombination, and omission studies. J Agric Food Chem. (2019) 67:4876–84. 10.1021/acs.jafc.9b01420 30920213

[18] Martins RC Lopes VV Silva Ferreira AC. Port wine oxidation management: A chemoinformatics approach. Am J Enol Viticult. (2009) 60:389A.

[19] Oliveira CM Barros AS Silva Ferreira AC Silva AMS. Influence of the temperature and oxygen exposure in red Port wine: A kinetic approach. Food Res Int. (2015) 75:337–47. 10.1016/j.foodres.2015.06.024 28454964

[20] Silva HOE Pinho PGD Machado BP Hogg T Marques JC Camara JS. Impact of forced-aging process on Madeira wine flavor. J Agric Food Chem. (2008) 56:11989–96. 10.1021/jf802147z 19053377

[21] Martins RC Monforte AR Silva Ferreira AC. Port wine oxidation management: A multiparametric kinetic approach. J Agric Food Chem. (2013) 61: 5371–9. 10.1021/jf4005109 23659499

[22] Castro CC Martins RC Teixeira JA Silva Ferreira AC. Application of a high-throughput process analytical technology metabolomics pipeline to Port wine forced ageing process. Food Chem. (2014) 143:384–91. 10.1016/j.foodchem.2013.07.138 24054256

[23] Monforte AR Jacobson D Silva Ferreira AC. Chemiomics: Network reconstruction and kinetics of Port wine aging. J Agric Food Chem. (2015) 63:2576–81. 10.1021/jf5055084 25671597

[24] Azevedo J Pinto J Teixeira N Oliveira J Cabral M Guedes de Pinho P. The impact of storage conditions and bottle orientation on the evolution of phenolic and volatile compounds of vintage Port wine. Foods. (2022) 11:2770. 10.3390/foods11182770 36140897 PMC9498223

[25] Ferreira IM Freitas F Pinheiro S Mourão MF Guido LF Gomes da Silva M. Impact of temperature during beer storage on beer chemical profile. LWT. (2022) 154:112688. 10.1016/j.lwt.2021.11268834500693

[26] Martínez A Vegara S Herranz-López M Martí N Valero M Micol V. Kinetic changes of polyphenols, anthocyanins and antioxidant capacity in forced aged hibiscus ale beer. J Inst Brew. (2017) 123:58–65. 10.1002/jib.387

[27] Pons-Mercadé P Giménez P Gombau J Vilomara G Conde M Cantos A. Oxygen consumption rate of lees during sparkling wine (Cava) aging; influence of the aging time. Food Chem. (2021) 342:128238. 10.1016/j.foodchem.2020.128238 33051100

[28] Ugliano M. Oxygen contribution to wine aroma evolution during bottle aging. J Agric Food Chem. (2013) 61:6125–36. 10.1021/jf400810v 23725213

[29] Isogai A Kanda R Hiraga Y Iwata H Sudo S. Contribution of 1,2-dihydroxy-5-(methylsulfinyl)pentan-3-one (DMTS-P1) to the formation of dimethyl trisulfide (DMTS) during the storage of Japanese sake. J Agric Food Chem. (2010) 58:7756–61. 10.1021/jf100707a 20527964

[30] Zhang XK Lan YB Huang Y Zhao X Duan CQ. Targeted metabolomics of anthocyanin derivatives during prolonged wine aging: Evolution, color contribution and aging prediction. Food Chem. (2021) 339:127795. 10.1016/j.foodchem.2020.127795 32836023

[31] Isogai A Kanda R Hiraga Y Nishimura T Iwata H Goto-Yamamoto N. Screening and identification of precursor compounds of dimethyl trisulfide (DMTS) in Japanese sake. J Agric Food Chem. (2008) 57:189–95. 10.1021/jf802582p 19090758

[32] Monforte AR Martins SIFS Silva Ferreira AC. Discrimination of white wine ageing based on untarget peak picking approach with multi-class target coupled with machine learning algorithms. Food Chem. (2021) 352:129288. 10.1016/j.foodchem.2021.129288 33677212

[33] Isogai A. Aroma compounds responsible for the aging of sake and their formation mechanism. J Society Brew Japan. (2009) 104:847–57. 10.6013/jbrewsocjapan.104.847

[34] Canas S Anjos O Caldeira I Belchior AP. Are the furanic aldehydes ratio and phenolic aldehydes ratios reliable to assess the addition of vanillin and caramel to the aged wine spirit? Food Control. (2019) 95:77–84. 10.1016/j.foodcont.2018.07.048

[35] Ma T Wang J Wang H Zhao Q Zhang F Ge Q. Wine aging and artificial simulated wine aging: Technologies, applications, challenges, and perspectives. Food Res Int. (2022) 153:110953. 10.1016/j.foodres.2022.111079 35227475

[36] Santos MC Nunes C Rocha MA Rodrigues A Rocha SM Saraiva JA. High pressure treatments accelerate changes in volatile composition of sulphur dioxide-free wine during bottle storage. Food Chem. (2015) 188:406–14. 10.1016/j.foodchem.2015.05.002 26041211

[37] Carvalho MJ Pereira V Pereira AC Pinto JL Marques JC. Evaluation of wine colour under accelerated and oak-cask ageing using CIELab and chemometric approaches. Food Bioprocess Technol. (2015) 8:2309–18. 10.1007/s11947-015-1585-x

[38] Del Alamo M Nevares I Gallego L Fernandez de Simon B Cadahia E. Micro-oxygenation strategy depends on origin and size of oak chips or staves during accelerated red wine aging. Anal Chim Acta. (2010) 660:92–101. 10.1016/j.aca.2009.11.044 20103149

[39] Caldeira I Vitória C Anjos O Fernandes TA Gallardo E Fargeton L. Wine spirit ageing with Chestnut Staves under different micro-oxygenation strategies: Effects on the volatile compounds and sensory profile. Appl Sci. (2021) 11:3991. 10.3390/app11093991

[40] Gao Y Hou L Gao J Li D Tian Z Fan B. Metabolomics approaches for the comprehensive evaluation of fermented foods: A review. Foods. (2021) 10:2294. 10.3390/foods10102294 34681343 PMC8534989

[41] Pinto J Oliveira AS Azevedo J Freitas VD Lopes P Roseira I. Assessment of oxidation compounds in oaked Chardonnay wines: A GC–MS and 1H NMR metabolomics approach. Food Chem. (2018) 257:120–7. 10.1016/j.foodchem.2018.02.156 29622187

[42] Arapitsas P Ugliano M Perenzoni D Angeli A Pangrazzi P Mattivi F. Wine metabolomics reveals new sulfonated products in bottled white wines, promoted by small amounts of oxygen. J Chromatogr A. (2016) 1429:155–65. 10.1016/j.chroma.2015.12.010 26709023

[43] Mu Y Su W Yu XT Mu YC Jiang L Wang HL. Untargeted metabolomics based on GC-TOF-MS reveals the optimal pre-fermentation time for black glutinous rice wine. Int J Food Prop. (2019) 22:2033–46. 10.1080/10942912.2019.1705481

[44] Jacobson D Monforte AR Silva Ferreira AC. Untangling the chemistry of Port wine aging with the use of GC-FID, multivariate statistics, and network reconstruction. J Agric Food Chem. (2013) 61:2513–21. 10.1021/jf3046544 23419138

[45] Fang C Du H Jia W Xu Y. Compositional differences and similarities between typical Chinese baijiu and western liquor as revealed by mass spectrometry-based metabolomics. Metabolites. (2018) 9:2. 10.3390/metabo9010002 30577624 PMC6358772

[46] Chen S Xu Y Qian MC. Aroma characterization of Chinese rice wine by gas chromatography-olfactometry, chemical quantitative analysis, and aroma reconstitution. J Agric Food Chem. (2013) 61:11295–302. 10.1021/jf4030536 24099139

[47] Qiu XJ. Influencing factors and formation mechanism of higher fatty acid ethyl ester in soy-flavor liquor. Stand Qual Light Ind. (2019) 163:78–80.

